# Non-excisional techniques for the treatment of intergluteal pilonidal sinus disease: a systematic review

**DOI:** 10.1007/s10151-023-02870-7

**Published:** 2023-11-06

**Authors:** E. A. Huurman, H. A. Galema, C. A. L. de Raaff, B. P. L. Wijnhoven, B. R. Toorenvliet, R. M. Smeenk

**Affiliations:** 1 Department of Surgical Oncology and Gastrointestinal Surgery, Erasmus MC Cancer Institute, Rotterdam, The Netherlands; 2 Department of Surgery, Albert Schweitzer Hospital, Dordrecht, The Netherlands; 3 Department of Surgery, Ikazia Hospital, Rotterdam, The Netherlands

**Keywords:** Pilonidal sinus, Pilonidal sinus disease, Minimally invasive procedures, Non-excisional techniques

## Abstract

Non-excisional techniques for pilonidal sinus disease (PSD) have gained popularity over the last years. The aim of this study was to review short and long-term outcomes for non-excisional techniques with special focus on the additive effect of treatment of the inner lining of the sinus cavity and the difference between primary and recurrent PSD. A systematic search was conducted in Embase, Medline, Web of Science Core Collection, Cochrane and Google Scholar databases for studies on non-excisional techniques for PSD including pit picking techniques with or without additional laser or phenol treatment, unroofing, endoscopic techniques and thrombin gelatin matrix application. Outcomes were recurrence rates, healing rates, complication rates, wound healing times and time taken to return to daily activities. In total, 31 studies comprising 8100 patients were included. Non-excisional techniques had overall healing rates ranging from 67 to 100%. Recurrence rates for pit picking, unroofing and gelatin matrix application varied from 0 to 16% depending on the follow-up time. Recurrence rates after additional laser, phenol and endoscopic techniques varied from 0 to 29%. Complication rates ranged from 0 to 16%, and the wound healing time was between three and forty-seven days. The return to daily activities varied from one to nine days. Non-excisional techniques are associated with fast recovery and low morbidity but recurrence rates are high. Techniques that attempt to additionally treat the inner lining of the sinus have worse recurrence rates than pit picking alone. Recurrence rates do not differ between primary and recurrent disease.

**Supplementary Information:**

The online version contains supplementary material available at 10.1007/s10151-023-02870-7.

## Introduction

Pilonidal Sinus Disease (PSD) of the intergluteal fold is a common disease with an estimated incidence of 25 per 100,000 [1]. It occurs primarily in young men and often requires surgical treatment [2]. There are many classification systems for PSD based on complexity of the disease. One may differentiate PSD between simple or complex disease as well as primary or recurrent PSD [3]. Different surgical techniques exist yet there is still no consensus on the optimal management of the disease.

Over the years non-excisional techniques have been developed to minimize surgical trauma and wound dehiscence and thus improve convalescence [4]. The general principle of these techniques is to remove hair and/or debris and debride the sinus cavity. Additional treatment of the inner lining of the sinus cavity using laser, phenol or endoscopic can be performed. Minimally  invasive techniques are fairly easy to apply and can be performed in an outpatient setting thereby lowering costs. Minimally invasive (non-excisional) approaches are less suited for complex or recurrent PSD, especially in an outpatient setting using local anesthesia. Some techniques have been used for a long time such as pit picking, pit picking with phenol application and unroofing. Relatively new techniques include laser treatment, endoscopic treatment and the application of a thrombin gelatin matrix. Previous reviews have never addressed the differences in outcomes between primary and recurrent disease.

The aim of this systematic review was  to provide an overview of non-excisional techniques and their outcomes for primary and recurrent PSD with special focus on the additive effect of treatment of the inner lining of the sinus cavity.

## Materials and methods

This systematic review was conducted according to the Preferred Reporting Items for Systematic Reviews and Meta-Analyses (see PRISMA 2020 checklist, Supplementary Table S1).

### Literature search and study selection

A systematic search was conducted by the Erasmus MC Medical Library in Embase, Medline, Web of Science Core Collection, Cochrane and Google Scholar databases. A search strategy was conducted using terms ‘pilonidal disease or sinus’ and ‘minimally invasive’. Minimally invasive techniques were defined as non-excisional techniques performed either in an outpatient clinic or operating room. We prefer to use the term ‘non excisional’ as it highlights the shared principle of these techniques in that the fibrotic sinus wall is left in situ. Supplementary File S1 shows the full search strings per database. The last search was conducted in March 13, 2023. Based on title and abstract, only clinical prospective studies concerning non-excisional or minimally invasive techniques in PSD surgery were included. Excluded were studies with less than 50 patients per study arm, retrospective studies, conference abstracts, studies published before 2000 and pediatric studies (patients< 14 years). Only journal articles written in English with full text available were considered. Articles were screened on full text for final inclusion. Articles that did not explicitly describe non-excisional techniques for PSD surgery were excluded. Screening on title and abstract and on full text was done by two authors independently (E.A.H. and H.A.G.). Disagreements were discussed with a third author (C.A.L.R.).

### Data extraction

The following data were extracted from tables and text: type of study; number of patients per study arm; patient characteristics including age, sex and type of PSD; type of non-excisional technique and patient-related outcomes (recurrence rates, healing rates, complication rates, wound healing times, time to return to daily activities and duration of follow-up). Healing rates were defined as complete closure of sinus and its tracts by subcutaneous scar formation and epithelization of the dermal pits.

### Quality assessment

Each study was assessed according to the level of evidence given by the Oxford Centre for Evidence-Based Medicine (OCEBM) study design [5]. 

## Results

The search identified 1478 articles. After removing duplicates, title and abstract screening was done on 782 articles. After full text screening 78 articles, 31 articles were included for qualitative assessment in the systematic review. The screening of references of included studies yielded no additional studies. Supplementary File S2 shows the PRISMA flow diagram. Included studies reported on the following non-excisional techniques: pit picking techniques, with or without additional laser or phenol treatment, unroofing, endoscopic techniques and the application of thrombin gelatin matrix.

### Pit picking

Three prospective studies were included [6–8]. A total of 1098 patients were diagnosed with primary PSD and 2682 with recurrent PSD (Table 1). Recurrence rates across studies ranged from 5.8% to 16.2% with a follow-up time of 12 to 120 months. Recurrence rates showed no statistically significant difference between primary and recurrent disease [6, 8]. Two studies reported that the time to return to daily activities ranged from one to three days [6, 7].

**Table 1:** Study characteristics and main outcomes of pit picking/gips procedure

First author (year)	CEBM	Study Design	Technique	Number of patients (*n*)	Primary versus recurrent (*n*)	Age(years)	Follow up in months	Complication *n* (%)	Primary healing, *n* (%)	Recurrence, *n* (%)	Healing rate after second treatment, *n* (%)	Time to daily activities (days)	Time to wound healing (weeks)
Di Castro (2016)	Level 3	Prospective cohort	Gips	2347	904 vs. 1443	19 (median)	16 (1–55) Median (range)	102 (4.3)	NA	137/2347 (5.8) Primary 59 (2.5) vs recurrent 78 (3.3)	NA	1 (0–16) Median (range)	4 (1–12) Median (range)
Colov (2011)	Level 3	Prospective cohort	Pit picking	75	34 vs. 41	30 (median)	12	11 (14.7)	NA	9/74 (12)	NA	3.2 (1–31) Mean (range)	3.5 (3.1–3.9) Median (range)
Gips (2008)	Level 3	Prospective cohort	Gips	1358	160 vs. 1198	20.9	120	22 (1.7)	1081/1358 (79.6)	189/1165 (16.2)	1119/1358 (82.4)	NA	3.4 ± 1.9

*NA* Not applicable, *CEBM* Centre for Evidence-Based Medicine. Data are mean ± SD, unless stated otherwise

### Unroofing

One prospective study including 203 patients with primary PSD was identified [9]. The recurrence rate was 4.9% with a median follow-up of 53 months. The complication rate was 4.4%. The median time to complete healing was 38 days. All patients were able to return to daily activities within six days.

### Endoscopic treatment

The seven studies on endoscopic treatment included six prospective series (Endoscopic pilonidal sinus treatment (EPSiT)) and one randomised controlled trial (Video-assisted ablation of pilonidal sinus (VAAPS) versus Bascom cleft lift) [10–16]. A total of 1579 patients were diagnosed with primary PSD and 231 with recurrent PSD (Table 2). In the randomised controlled trial by Milone et al. the recurrence rate after VAAPS was 3.9% after 12 months and 24.3% at five years. Recurrence rates across the cohort studies varied between 0% and 13.5% with a follow-up ranging from 12 to 56 months. No statistically significant difference in recurrence rate and primary healing rate was observed between primary and recurrent disease [14, 15]. Five studies reported a time to return to daily activities ranging from one to six days [10–13, 15].

**Table 2:** Study characteristics and main outcomes of endoscopic treatment

First author (year)	CEBM	Study design	Technique	Number of patients (*n*)	Primary versus recurrent (*n*)	Age(years)	Follow up in months	Complication *n* (%)	Primary healing, *n* (%)	Recurrence, *n* (%)	Healing rate after second treatment, *n* (%)	Time to daily activities (days)	Time to wound healing (days/weeks)
Milone (2020)	Level 2	Randomised controlled trial	VAAPS (vs. BCL)	74 (vs 67)	74 vs. 0	25.5	60	4 (5.2)	NA	18/74 (24.3)	NA	1.8 ± 1.2	NA
Azhough (2021)	Level 3	Prospective cohort	EPSiT	100	100 vs. 0	27.1	14.3 ± 2.4	0 (0)	NA	4/100 (4)	NA	2–5	2–4 weeks
Giarratano (2017)	Level 3	Prospective cohort	EPSiT	77	68 vs. 9	23 (median)	25 (17–40) Median (range)	0 (0)	71/77 (92)	4/77 (5)	74/77 (96)	6 ± 3 (2–14) Mean ± SD (range)	26 (15–45) days Median (range)
Hinksman (2022)	Level 3	Prospective cohort	EPSiT	137	65 vs. 72	26.3	56.2 ± 17.1	2 (1.5)	91/126 (72.2)	17/126 (13.5)	109/146 (75)	2.8 (1–5) Mean (range)	NA
Kalaiselvan (2020)	Level 3	Prospective cohort	EPSiT	74	33 vs. 41	21 (median)	13 (2–114) Median (range)	2 (2.7)	44/74 (67)	0 (0)	51/66 (77) Primary 28/31 (90) vs. recurrent 29/35 (83)	NA	NA
Meinero (2016)	Level 3	Prospective cohort	EPSiT	250	141 vs. 109	24.3	12	0 (0)	237/250 (94.8)	12/237 (5.1) Primary 6/141 (4.2) vs recurrent 6/109 (5.5)	246/250 (98.4)	2 ± 0.5	26.7 ± 10.4 days
Mendes (2019)	Level 3	Prospective cohort	EPSiT	67	NA	31	NA	5 (7)	(91)	6/67 (9)	NA	NA	4 (3–12) weeks Mean (range)

*NA* Not applicable, *VAAPS* video-assisted ablation of pilonidal sinus, *BCL* bascom cleft lift, *EPSiT* endoscopic pilonidal sinus treatment, *CEBM* Centre for Evidence-Based Medicine. Data are mean ± SD, unless stated otherwise

### Laser treatment (SilaC, SiLaT and PiLaT)

There were four prospective cohort studies on laser treatment and one randomised controlled trial (PiLat versus Limberg Flap) [17–21]. A total of 788 patients were diagnosed with primary PSD and 120 with recurrent PSD (Table 3). In the randomised controlled trial by Dalbasi et al., recurrence rate was 4% with a follow-up of only two months. All patients were able to return to daily activities within three days. Recurrence rates of the cohort studies ranged between 1.6% and 26.4% with a follow-up time ranging from 10 to 17 months. One study found all recurrences in the primary treatment group [20]. In all studies, the time to return to daily activities ranged from one and six days.

**Table 3:** Study characteristics and main outcomes of laser treatment

First author (year)	CEBM	Study Design	Technique	Number of patients (*n*)	Primary versus recurrent (*n*)	Age(years)	Follow up in months	Complication, *n* (%)	Primary healing, *n* (%)	Recurrence, *n* (%)	Healing rate after second treatment, *n* (%)	Time to daily activities (days)	Time to wound healing (days/weeks)
Dalbasi (2020)	Level 2	Randomised controlled trial	PiLat (vs LF)	100 (vs 100)	100 vs. 0	26.9	2	0 (0)	NA	4/100 (4)	NA	2.3 ± 0.5	NA
Dessily (2019)	Level 3	Prospective cohort	SiLaC	200	200 vs. 0	24.5	17 ±8.7	19 (9.5)	188/200 (94)	22/144 (15.2)	197/200 (98.5)	4–11	19.5 ± 14.4 days
Georgiou (2018)	Level 3	Prospective cohort	PiLaT	60	60 vs. 0	22.7	12	1 (1.6)	55/60 (92)	1/60 (1.6)	59/59 (100)	< 3	25.4 (17–40) days Median (range)
Pappas (2018)	Level 3	Prospective cohort	SiLaT	237	210 vs. 27	24 (median)	12 (median)	17 (7.2)	214/237 (90.3)	7/237 (3.3) Primary 7 (3.3) vs. recurrent 0 (0)	232/237 (97.9)	0–2	47 (30–70) days Median (range)
Sluckin (2022)	Level 3	Prospective cohort	SiLaC	311	218 vs. 93	27.3	10 (1–52) Median (range)	16 (5.1)	206/311 (66.2)	82/311 (26.4)	287/311 (92.2)	6 (0–42) Mean (range)	6 (1–24) weeks Mean (range)

NA Not applicable, *PiLat* pilonidal disease laser treatment, *LF* limberg flap, *SiLaC* sinus laser closure, *CEBM* Centre for Evidence-Based Medicine. Data are mean ± SD, unless stated otherwise

### Crystallized phenol

Of the fourteen included studies, ten were prospective series and four were randomised controlled trials [22–36]. A total of 2055 patients were diagnosed as primary PSD and the other 275 as recurrent PSD (Table 4). First, the outcomes of the randomised controlled trials will be described. In the randomised controlled trial by Calikoglu et al. the recurrence rate was 18.6% with a mean follow-up of 38 months. All patients were able to return to daily activities within three days. In the randomised controlled trial by Emiroglu et al. the recurrence rate was 2% in the phenol 30% group compared to 3.9% in the phenol 80% group with a mean follow-up time of 12 months. All patients were able to return to daily activities within four days. In the randomised controlled trial by Lopez et al. no recurrence was reported with a mean follow-up of 16 months. The mean length of sick leave was 20 days. In the randomised controlled trial by Sevinç et al. the recurrence rate was 12% with a mean follow-up of 41 months. The remaining 10 cohort studies reported on the outcomes of phenol treatment only. Recurrence rates across these studies ranged between 2% and 28.9% with a follow-up of six to 60 months. Four studies showed a time to daily activities between one and nine days.

**Table 4:** Study characteristics and main outcomes of Crystallized phenol application

First author (year)	CEBM	Study type	Technique	Number of patients (*n*)	Primary versus recurrent (*n*)	Age (years)	Follow up in months	Complication *n* (%)	Primary healing, *n* (%)	Recurrence, *n* (%)	Healing rate after second/third treatment, *n* (%)	Time to daily activities (days)	Time to wound healing (days/weeks)
Calikoglu (2017)	Level 2	Randomised controlled trial	Phenol (vs open)	70 (vs 70)	58 vs. 12	30.1	38.3 ± 11.3	6 (8.6)	NA	13/70 (18.6)	NA	0.8 ± 2.8	16.2 ± 8.7 days
Emiroglu (2016)	Level 2	Randomised controlled trial	Phenol 30%	49	51 vs. 8	25	11.9 (4–20) Mean (range)	3 (6.1)	37/49 (75.5)	1/49 (2)	39/49 (79.6)	2.2 (0–4) Median (range)	3.9 (2–6) weeks Median (range)
Emiroglu (2016)	Level 2	Randomised controlled trial	Phenol 80%	52	45 vs. 7	24	12.1 (4–20) Mean (range)	4 (7.7)	43/52 (82.7)	2/52 (3.9)	45/52 (86.5)	2.7 (0–4) Median (range)	3.7 (2–7) weeks Median (range)
Lopez (2023)	Level 2	Randomised controlled trial	Phenol (vs conventional surgery)	60 (vs 56)	60 vs. 0	24.4	16	3 (5)	NA	0 (0)	NA	19.6 ± 3.8	NA
Sevinç (2022)	Level 2	Randomised controlled trial	Phenol	100	90 vs. 10	24.6	41.3 + 3.2	0 (0)	53 (53)	12 (12)	94 (94)	NA	10 (5–42) days Median (range)
Dag (2012)	Level 3	Prospective cohort	Phenol	76	76 vs. 0	24.3	25 (13–48) Mean (range)	12 (15.7)	46/76 (60.5)	1/76 (2)	51/76 (67)	0	16 (10–45) days Mean (range)
Dogru (2020)	Level 3	Prospective cohort	Phenol	1026	1026 vs. 0	26.9	46.9 ± 32.3	0 (0)	806/1026 (78.6)	220/1026 (21.4)	865/1026 (84.3)	NA	8.9 ± 7.9 days
Kargin (2022)	Level 3	Prospective cohort	Phenol	190	0 vs. 190	26.3	60	NA	85/190 (44.7)	55/190 (28.9)	136/190 (71.5)	NA	NA
Kaymakcioglu (2005)	Level 3	Prospective cohort	Phenol	143	143 vs. 0	26.3	24	23 (16)	NA	12/143 (8.3)	NA	NA	NA
Olmez (2013)	Level 3	Prospective cohort	Phenol	83	68 vs. 15	26.6	25.7 ± 8.5	8 (10)	74/83 (89.2)	2/83 (2.5)	NA	NA	28.5 ± 14.9 days
Ozturk (2019)	Level 3	Prospective cohort	Phenol	67	59 vs. 8	26.8	27.3 (6–37) Median (range)	5 (7.4)	NA	3/67 (4.4)	NA	NA	38 days
Sakcak (2010)	Level 3	Prospective cohort	Phenol 40%	54	49 vs. 5	28.4)	32.4 (12-48)	2 (1.7)	NA	4/54 (7.4)	NA	3.1 (0–14) Mean (range)	30.5 (11–6) Mean (range)
Sakcak (2010)	Level 3	Prospective cohort	Phenol 80%	58	51 vs. 7	27.1	34.8 (12-48)	10 (8.9)	NA	9/58 (15.5)	NA	8.6 (0–42) Mean (range)	37 (18–88) Mean (range)
Sozuer (2020)	Level 3	Prospective cohort	Phenol	209	196 vs. 13	25.5	12	6 (2.8)	187/209 (89.3)	17/209 (8.1)	196/209 (93.7)	NA	NA
Tazeoglu (2022)	Level 3	Prospective cohort	Phenol	83	83 vs. 0	24.4	18.2 ± 3.6	NA	NA	7/83 (8.4)	NA	3 ± 1.7	NA
Yuksel ME. (2017)	Level 3	Prospective cohort	Phenol	50	NA	27	6	4 (8)	(88)	4/50 (8)	NA	NA	30 (range 13–50) Mean (range)

*NA* Not applicable, *PP* pit picking, *CEBM* Centre for Evidence-Based Medicine. Data are mean ± SD, unless stated otherwise

### Thrombin gelatin matrix application

The study by Elbanna et al. investigated the effect of thrombin gelatin matrix as a new sealant for the treatment of PSD [37]. Of these, 47 patients were diagnosed as primary PSD and three as recurrent PSD. The median age was 22 years. Recurrence rate was 4% with a median follow-up of 24 months. For 94% of the patients the wound was completely healed within 2 weeks. All patients were able to return to daily activities within two days.

### Outcomes of non-excisional techniques

In total, 31 studies comprising 8100 patients were analysed. These studies showed that non-excisional techniques had overall healing rates ranging from 67 to 100%. Recurrence rates varied from 0 to 29% depending on the follow-up time. Higher recurrence rates were observed with shorter follow-up times following supplementary treatment of the inner lining of the sinus cavity. Complication rates ranged from 0 and 16%, and the wound healing time was between six and 47 days. Laser and phenol have longer reported healing times compared to pit picking only. Complication rates were similar. The return to daily activities varied from one to nine days. Table 5 shows the characteristics of the studies and their main outcomes.

**Table 5:** Study characteristics and overall outcomes of non-excisional techniques

	PP/gips	Unroofing	Endoscopic	Laser	Phenol	TGM
Number of patients, *n*	3780	203	779	908	2380	50
Number of studies, *n*	3	1	7	5	14	1
Recurrence rate (%)	5.8–16.2	4.9	0–24.3	1.6–26.4	2–28.9	4.0
Follow up time (months)	12–120	53	12–60	2–17	6–60	24
Complication rate (%)	1.7–14.7	4.4	0–7	1.6–9.5	0–16	2
Healing rate (%)	79.6	NA	67–94.8	66.2–100	44.4–89.3	94
Second healing rate (%)	82.4	NA	75–98.4	92.2–100	67–94	NA
Healing time (days)	21–28	38	14–28	6–47	3–38	NA
Return to daily activities (days)	1–3	3	1–6	1–6	1–9	2

*NA* Not applicable, *TGM* thrombin gelatin matrix, *PP* pit picking. Data presented are within a range, unless stated otherwise

## Discussion

This review reports the short- and long-term outcomes of non-excisional techniques for primary and recurrent PSD including the effect of treatment of the inner lining of the sinus cavity. An advantage of non-excisional techniques is a quick recovery and low complication rate. Although recurrence rates are high, patients may consent to a procedure that induces minimal surgical trauma, has a low morbidity, swift recovery and fast return to daily activities. These factors may be more important for younger patients that wish to resume study or work quickly. The choice for a non-excisional technique for primary PSD as a first line treatment therefore seems reasonable. This review aimed to examine whether there are any reports showing that non-excisional techniques are also applicable for recurrent PSD. Only five studies reported separately on the outcomes of non-excisional techniques for recurrent disease [6, 8, 14, 15, 20]. Four studies showed no difference in recurrence rates [6, 8, 14, 15, 20]. One study indicated no difference in primary healing rates [14]. Other outcomes such as morbidity and recovery time were not separately described. Taking this into account, one cannot draw conclusions from the available studies.

At present, radical excision with secondary wound healing is still widely used irrespective of disease complexity [38, 39]. However, morbidity is high and time to complete wound healing is long, leading to a delayed return to daily activities. Moreover, the 5-year recurrence rate is between 16 and 22% which further increases to 44% after 10 years [40–43]. Secondary intention wound healing may impact quality of life, healthcare costs and costs from a societal perspective due to absence from work [44, 45]. Off-midline closure techniques such as the Bascom cleft lift and the Karydakis flap have lower recurrence rates (1.9% to 10.2%) [40]. Despite this advantage, both techniques have not been implemented on a wider scale, most likely due to the complexity and associated learning curve. Some advocate the use of flap techniques only for complex or recurrent PSD [46].

According to the data of this review, the overall recurrence rates for non-excisional techniques for PSD are high, and they vary depending on the duration of follow-up. Although most recurrences present within five years, they may occur even decades after treatment. This was already reported by Stauffer et al. in 2018, and it was also stated by Doll et al [40, 41]. 

In order to further improve recurrence outcomes of non-excisional techniques, some surgeons treat the inner lining of the sinus cavity after pit-picking and debridement with laser, phenol or endoscopic inspection and cautery. Based on the data presented in our review, it appears that they do not reduce recurrence rates; instead, they increase them. Additionally, the healing times after laser and phenol treatement seem to be even longer than after pit picking alone. The use of phenol may also carry a health hazard for patients and health care personnel [47]. Finally, laser and endoscopic treatment is much more expensive than pit picking alone. Taking all of this into account, treating the inner lining of the sinus cavity should perhaps be discontinued.

When consenting patients with PSD to a non-excisional technique, the higher risk of recurrence in the long term should be discussed against the short-term benefits. During shared decision making, a step-up algorithm may be useful whereby a non-excisional technique is performed once, and in the case of a recurrence a more complex second line treatment (flap technique) is performed in order to prevent another recurrence. The Dutch guideline on PSD supports this view [46]. 

This review has its limitations. The included studies, although prospective in design, still suffer from selection bias, incomplete reporting on outcomes, short follow up and there is no uniform classification system for severity of disease used. Some authors have combined techniques, making it more difficult to analyse the literature. Also, there is no uniform definition of recurrence. The included studies are clinically heterogenous and therefore conclusions need to be carefully interpreted. Future studies need well-defined disease classifications and definitions to accurately elucidate the outcomes of non-excisional techniques for primary and recurrent disease. Additionally, RCTs are needed to investigate the effectiveness of treatment of the inner lining of the sinus cavity.

## Conclusion

The current systematic review shows that non-excisional techniques are associated with fast recovery and low morbidity but recurrence rates are high. Techniques that attempt to additionally treat the inner lining of the sinus have worse recurrence rates than pit picking alone. Recurrence rates do not differ between primary and recurrent disease.

### Supplementary Information

Below is the link to the electronic supplementary material.

Supplementary file1 (PDF 15 KB)

Supplementary file2 (DOCX 51 KB)

## Data Availability

All data generated or analysed during the current study are included in this published article (and its supplementary information files).
