# Supplementary material for: Non-excisional techniques for the treatment of intergluteal pilonidal sinus disease: a systematic review
Source: Tech Coloproctol. 2023 Nov 6;27(12):1191–200. doi: 10.1007/s10151-023-02870-7 (PMC10638206; doi:10.1007/s10151-023-02870-7)
Supplement: Supplementary file 1 — Supplementary file1 (PDF 15 KB) [file 10151_2023_2870_MOESM1_ESM.docx]

20230312 Noor Huurman

Pilonidal sinus minimally invasive

| **Database searched** | **via** | **Years of coverage** | **Records** | **Records after duplicates removed** |
| --- | --- | --- | --- | --- |
| Embase | Embase.com | 1971 - Present | 588 | 577 |
| Medline ALL | Ovid | 1946 - Present | 382 | 61 |
| Web of Science Core Collection* | Web of Knowledge | 1975 - Present | 449 | 124 |
| Cochrane Central Register of Controlled Trials | Wiley | 1992 - Present | 59 | 20 |
| **Total** | | | **1478** | **782** |

*Science Citation Index Expanded (1975-present) ; Social Sciences Citation Index (1975-present) ; Arts & Humanities Citation Index (1975-present) ; Conference Proceedings Citation Index- Science (1990-present) ; Conference Proceedings Citation Index- Social Science & Humanities (1990-present) ; Emerging Sources Citation Index (2015-present)

***new references:*** 151*

**Compared to the search performed on 12 October 2021. Not with update from June 2022.*

**Embase.com**

('pilonidal sinus'/de OR (((pilonidal* OR pilonidal* OR sacrococcyg* OR barber-hair OR coccygeal OR hair-bearing) NEAR/3 (sinus* OR cyst* OR disease* OR fistula* OR excision*))):ab,ti OR (p-sinus OR psd):ti) AND ('minimally invasive procedure'/exp OR sinusectomy/de OR endoscopy/exp OR 'endoscopic surgery'/exp OR 'endoscopic therapy'/de OR 'ablation therapy'/exp OR laser/exp OR 'laser surgery'/exp OR 'laser therapy'/exp OR deroofing/de OR 'fibrin glue'/de OR 'caustic agent'/exp OR 'phenol derivative'/exp OR radiosurgery/exp OR sinotomy/de OR (mini*-invasi* OR less*-invasi* OR sinusectom* OR sinotom* OR Gips* OR endoscop* OR EPSiT* OR ablation* OR VAAPS* OR laser* OR Bascom-1* OR Pit-picking* OR deroofing* OR unroofing* OR FiLaC* OR siLaC* OR rfa OR ((rf OR Radiofrequenc* OR minimal*) NEAR/3 (excis* OR surger*)) OR fibrin-glue OR silver-nitrat* OR phenol* OR radiosurg*):Ab,ti) NOT ([conference abstract]/lim AND [2000-2019]/py) NOT ([animals]/lim NOT [humans]/lim) AND [english]/lim

**Medline ALL Ovid**

(Pilonidal Sinus/ OR (((pilonidal* OR pilonidal* OR sacrococcyg* OR barber-hair OR coccygeal OR hair-bearing) ADJ3 (sinus* OR cyst* OR disease* OR fistula* OR excision*))).ab,ti. OR (p-sinus OR psd).ti.) AND (exp Minimally Invasive Surgical Procedures/ OR exp Endoscopy/ OR exp Laser Therapy/ OR exp Lasers/ OR Fibrin Tissue Adhesive/ OR exp Radiosurgery/ OR (mini*-invasi* OR less*-invasi* OR sinusectom* OR sinotom* OR Gips* OR endoscop* OR EPSiT* OR ablation* OR VAAPS* OR laser* OR Bascom-1* OR Pit-picking* OR deroofing* OR unroofing* OR FiLaC* OR siLaC* OR rfa OR ((rf OR Radiofrequenc* OR minimal*) ADJ3 (excis* OR surger*)) OR fibrin-glue OR silver-nitrat* OR phenol* OR radiosurg*).ab,ti.) NOT (exp animals/ NOT humans/) AND english.lg.

**Web of Science Core Collection***

TS=(((((pilonidal* OR pilonidal* OR sacrococcyg* OR barber-hair OR coccygeal OR hair-bearing) NEAR/2 (sinus* OR cyst* OR disease* OR fistula* OR excision*))) OR (p-sinus OR psd):ti) AND ((mini*-invasi* OR less*-invasi* OR sinusectom* OR sinotom* OR Gips* OR endoscop* OR EPSiT* OR ablation* OR VAAPS* OR laser* OR Bascom-1* OR Pit-picking* OR deroofing* OR unroofing* OR FiLaC* OR siLaC* OR rfa OR ((rf OR Radiofrequenc* OR minimal*) NEAR/2 (excis* OR surger*)) OR fibrin-glue OR silver-nitrat* OR phenol* OR radiosurg*)))

**Cochrane Central Register of Controlled Trials**

(((((pilonidal* OR pilonidal* OR sacrococcyg* OR barber NEXT hair OR coccygeal OR hair NEXT bearing) NEAR/2 (sinus* OR cyst* OR disease* OR fistula* OR excision*))) OR (p NEXT sinus OR psd):ti) AND ((mini* NEXT invasi* OR less* NEXT invasi* OR sinusectom* OR sinotom* OR Gips* OR endoscop* OR EPSiT* OR ablation* OR VAAPS* OR laser* OR Bascom NEXT 1* OR Pit NEXT picking* OR deroofing* OR unroofing* OR FiLaC* OR siLaC* OR rfa OR ((rf OR Radiofrequenc* OR minimal*) NEAR/2 (excis* OR surger*)) OR fibrin NEXT glue OR silver NEXT nitrat* OR phenol* OR radiosurg*)))
