# Supplementary material for: Non-excisional techniques for the treatment of intergluteal pilonidal sinus disease: a systematic review
Source: Tech Coloproctol. 2023 Nov 6;27(12):1191–200. doi: 10.1007/s10151-023-02870-7 (PMC10638206; doi:10.1007/s10151-023-02870-7)
Supplement: Supplementary file 2 — Supplementary file2 (DOCX 51 KB) [file 10151_2023_2870_MOESM2_ESM.docx]

**Identification of studies via databases and registers**

Records removed *before screening*:

Duplicate records removed (n = 696)

Records identified from*:

Databases (n = 1478)

**Identification**

Records screened

(n = 782)

Records excluded**

(n = 704)

Reports not retrieved (n = 6):

- Full text not available (n = 3)
- Full text in German (n = 3)

Reports sought for retrieval

(n = 78)

**Screening**

Reports excluded (n = 41):

- Conference abstracts (n = 14)
- Articles on minimal excision (n = 5)
- Article on pilonidal abscess (n = 1)
- Studies with fewer than 50 patients per study arm (n = 8)
- Articles involving children (n = 2)
- Retrospective articles (n = 7)
- Studies with no outcomes of interest (n = 2)
- Studies with the same patient population (n = 2)

Reports assessed for eligibility

(n = 72)

Studies included in review

(n = 31)

**Included**

*Consider, if feasible to do so, reporting the number of records identified from each database or register searched (rather than the total number across all databases/registers).

**If automation tools were used, indicate how many records were excluded by a human and how many were excluded by automation tools.

*From:*  Page MJ, McKenzie JE, Bossuyt PM, Boutron I, Hoffmann TC, Mulrow CD, et al. The PRISMA 2020 statement: an updated guideline for reporting systematic reviews. BMJ 2021;372:n71. doi: 10.1136/bmj.n71

For more information, visit: <http://www.prisma-statement.org/>
